# In vitro inhibitory effects of *Hypoxis hemerocallidea* and *Pentanisia prunelloides* on α-amylase, α-glucosidase and pancreatic lipase activities associated with obesity-related metabolic disorders

**DOI:** 10.1186/s12906-026-05458-4

**Published:** 2026-07-18

**Authors:** Mlungisi F. Mngoma, Lembe S. Magwaza, Asanda Mditshwa, Samson Z. Tesfay, Blessing N. Mkhwanazi

**Affiliations:** 1 Discipline of Horticultural Science, School of Agriculture and Science, University of KwaZulu-Natal, Private Bag X01, Scottsville, Pietermaritzburg, 3209 South Africa; 2 Postharvest Research Group, Cranfield University, Cranfield, MK43 0AL UK; 3 South African–German Bilateral Research Chair in Resilient Food Systems, Faculty of Agriculture and Natural Sciences, University of Mpumalanga, Mbombela, 1200 South Africa; 4 Discipline of Dietetics and Nutrition, School of Agriculture and Science, University of KwaZulu-Natal, Private Bag X01, Scottsville, Pietermaritzburg, 3209 South Africa

**Keywords:** Digestive enzymes, Inhibition, Anti-obesity, Metabolic disorder, Plant metabolites

## Abstract

Obesity is a complex metabolic disorder associated with dysregulated lipid metabolism and impaired adipose tissue function, resulting in the development of various chronic diseases such as diabetes mellitus. Plant-based therapeutic strategies, targeting the inhibition of digestive enzymes, have gained increasing attention as potential safe alternatives to synthetic drugs. This study investigated the inhibitory effect of root and leaf extracts of *Hypoxis hemerocallidea* Fisch., C.A.Mey. & Avé-Lall. and *Pentanisia prunelloides* (Klotzsch ex Eckl. & Zeyh.) Walp, against α-amylase, α-glucosidase, and pancreatic lipase, and related these activities to metabolite profiles to explore their potential relevance in obesity-associated metabolic dysfunctions. The results showed that *H. hemerocallidea* leaves exhibited a strong inhibitory activity against pancreatic lipase (98.68%) and α-glucosidase (98.10%), while α-amylase was considerably lower (11.71%). In *P. prunelloides*, both roots and leaves showed a strong α-glucosidase inhibitory activity of 99.02% and 96.38%, respectively. Moderate α-Amylase inhibitory activity was observed in the roots of *H. hemerocallidea* (53.14%), and *P. prunelloides* (50.63%), with the inhibition levels comparable to the lipase standard inhibitor acarbose (55.65%). Metabolite profiling using liquid chromatography-mass spectrometry (LC-MS) analysis revealed diverse putatively identified bioactive metabolites, including flavonoids, phenolic acids, phenolic glycosides, proanthocyanidins, saponins, terpenoids, and xanthones. Correlation and multivariate analyses suggested potential associations between metabolite abundance and enzyme inhibitory activities. Collectively, these findings suggest that both plant species possess substantial metabolite diversity and promising in vitro enzyme inhibitory activities, with *H. hemerocallidea* exhibiting comparatively stronger inhibitory activity. However, no compound isolation, cytotoxicity assessment, and in vivo validation was performed; therefore, the findings should be considered preliminary and require further pharmacological and mechanistic investigation.

**Supplementary Information:**

The online version contains supplementary material available at https://doi.org/10.1186/s12906-026-05458-4.

## Introduction

Obesity is an intricate metabolic disorder characterised by endocrine dysfunction, which plays a crucial role in the development of various chronic diseases [1]. One of the key metabolic disturbances linked with obesity is the alteration of lipid metabolism and lipoproteins dysfunction, leading to elevated free fatty acid concentration in the bloodstream [2]. Additionally, obesity-induced metabolic syndrome is closely related to structural and functional alterations in adipose tissues. Beyond its role as a storage site for excess lipids, adipose tissue functions as an endocrine organ that secretes a variety of adipokines and signalling molecules that regulate metabolism and insulin sensitivity [3]. However, adipocyte hypertrophy in obesity can lead to hypoxic conditions, which triggers the expression of pro-inflammatory cytokines such as tumour necrosis factor-alpha (TNF-α) and interleukin-6 (IL-6) [4]. Hypoxia also reduces the secretion of beneficial adipokines such as adiponectin, known for its anti-inflammatory properties and insulin-sensitising effects, while promoting elevated levels of adipokines such as leptin and resistin, which, when dysregulated, contribute to insulin resistance [5]. These disturbances in adipose tissue and insulin signalling impair glucose transport, lipolysis, and glycogen, ultimately contributing to the development of chronic diseases such as dyslipidemia, tumours, type 2 diabetes, and cardiovascular conditions [6].

Current management strategies for obesity include lifestyle modification, pharmacotherapy, and bariatric surgery. Among these approaches, pharmacological interventions targeting digestive enzymes involved in lipid and carbohydrate metabolism have gained considerable attention as complementary strategies for obesity management and associated metabolic disorders. These interventions primarily focus on inhibiting pancreatic lipase, α-glucosidase, and α-amylase which are responsible for the digestion and absorption of dietary fats and carbohydrates [7]. Pancreatic lipase, secreted by the pancreas and transported to the duodenum, plays a critical role in the digestion of triacylglycerols into free fatty acids and monoacylglycerols for absorption in the intestine [8]. Therefore, blocking this enzyme reduces fat absorption and contribute to weight management [9]. Clinical studies involve orlistat, a pancreatic lipase inhibitor, demonstrated reduced dietary fat absorption and increased faecal fat excretion, leading to greater weight loss in obese patients [10, 11]. Similarly, α-glucosidase, a brush border enzyme located in the small intestine, catalyses the hydrolysis of oligosaccharides and disaccharides into absorbable glucose molecule [12]. Excessive glucose release from dietary carbohydrates contributes to postprandial hyperglycaemia, increased caloric availability, and lipid accumulation, which are closely associated with obesity and metabolic syndrome. Inhibiting this enzyme delays carbohydrate digestion, and glucose absorption, thereby assisting in the regulation of postprandial glucose levels and obesity-associated metabolic complications [13]. In addition, α-amylase which is prominent in saliva and pancreatic juices, hydrolyses insoluble starch molecules into smaller oligosaccharides such as maltose and dextrins [14]. Inhibiting this enzyme may reduce carbohydrate digestion and glucose uptake, thereby contributing to improved metabolic regulation and reduced risk of obesity-associated hyperglycaemia [15].

In recent years, increasing attention has been directed towards the development of plant-derived therapeutic agents targeting digestive enzymes involved in obesity and related metabolic disorders. Medicinal plants offer a promising alternatives to synthetic drugs due to their diverse phytochemical composition and potential to modulate lipid and carbohydrate metabolism with fewer adverse effects [16]. Several plant species demonstrated inhibitory activities against pancreatic lipase, α-glucosidase, and α-amylase, with phenolic compounds implicated as key contributors to these biological activities. However, despite growing interest in medicinal plants as natural interventions for obesity-associated metabolic disorders, the mechanisms underlying their metabolic effects remain poorly understood. Particularly, *H. hemerocallidea* a perennial herb characterised by strap-like leaves and yellow star-shaped flowers, and *P. prunelloides*, recognised by its tuberous roots and purple-blue flowers, are medicinal plants widely utilised in traditional African medicine for the management of various ailments, including inflammation, diabetes, immune-related disorders, and gastrointestinal complications [17, 18]. Most of these complications are closely associated with obesity-related metabolic dysfunction. Previous studies using male Sprague-Dawley rat models have reported the potential metabolic relevance of *Hypoxis hemerocallidea*, including effects on body weight regulation under diabetic conditions [19]. Similarly, Makhubu et al. [20] have reported the antidiabetic potential of *Pentanisia prunelloides* through inhibition of α-amylase and α-glucosidase, together with notable antioxidant activity, supporting its traditional use in the management of diabetes and related metabolic disorders. Despite these findings, limited information is available regarding the inhibitory effects of these plant species and their respective plant parts on key digestive enzymes involved in lipid and carbohydrate metabolism. Therefore, the present study aimed to evaluate the inhibitory effects of *H. hemerocallidea* and *P. prunelloides* extracts against pancreatic lipase, α-glucosidase, and α-amylase, and to relate these activities to their metabolite profiles using LC-MS-based metabolomic analysis in the context of obesity-associated metabolic disorders.

## Materials and methods

### Plant collection and extraction

Samples of *H. hemerocallidea* and *P. prunelloides* were harvested from the University of KwaZulu-Natal (UKZN) Botanical Garden, Pietermaritzburg, South Africa (29°37’28” S30°24’13” E). The botanical garden is institutional property; therefore, sampling was conducted on private institutional land rather than on public or protected land, and no additional governmental permits or collection licences were required. Permission for collecting samples was obtained from the responsible horticulturist before sampling. Plant identification was confirmed by a qualified horticulturist and botanist at UKZN, Mrs Alison Young. The botanical names were authenticated as *Hypoxis hemerocallidea* Fisch., C.A.Mey. & Avé-Lall. and *Pentanisia prunelloides* (Klotzsch ex Eckl. & Zeyh.) Walp. Voucher specimens were deposited at the Bews Herbarium, School of Life Sciences, UKZN, Pietermaritzburg, South Africa, under voucher specimen numbers MM1 (*P. prunelloides*) and MM2 (*H. hemerocallidea*). The samples were separated into roots and leaves and rinsed under running water to remove debris before being dried in an Incotherm oven (Laboratory Consumables Pty, South Africa) at a constant temperature of 60 °C for 72 h, following a method described by Ncube et al. [21]. The dried plant materials were subsequently ground into a fine homogeneous powder using an electric Waring blender and stored in airtight containers at room temperature until extraction.

The dried plant material was extracted separately by macerating 100 g of finely powdered sample in 200 mL of 70% (v/v) methanol–distilled water solution [22]. The mixtures were shaken on a Labcorn platform shaker (Laboratory Consumables, PTY, Durban, South Africa) at 150 rpm at a temperature of 25 °C for 2 days. The plant extracts were centrifuged at 3000 rpm for 5 min and filtered through Whatman No. 1 filter paper to obtain homogeneous extracts. The filtrates were subsequently concentrated using a rotary evaporator under reduced pressure at 35 °C and further air-dried in a fume hood until complete solvent evaporation was achieved. The dried crude extracts were stored at -80 °C deep freezer until further analysis to preserve their stability and phytochemical integrity. Prior to enzyme inhibition assays, crude extracts were reconstituted to a stock concentration of 1 mg/mL in dimethyl sulfoxide (DMSO), with the final DMSO concentration maintained below 1% in all assays to avoid interference with enzyme activity.

### Pancreatic lipase inhibitory activity of plant extracts

The activity of plant extracts as inhibitors of pancreatic lipase (EC 3.1.1.3) was measured using a modified method described by Marrelli et al. [23]. The solution was prepared by dissolving porcine pancreatic lipase in dimethylsulfoxide (DMSO) at a concentration of 0.2 mg/mL (2 U/mL). The enzyme activity was prepared fresh prior to analysis and maintained on ice during assay preparation to preserve enzymatic activity. A 2mM p-Nitrophenyl butyrate (pNPB) solution was prepared in DMSO. The reaction mixture was set up in a 96 well plate by adding 140 µL of 20 mM Tris-HCl buffer (pH 8.5), followed by 20 µL of plant extract and 20 µL of porcine pancreatic lipase enzyme. Plant extracts were tested at a final concentration of 0.25 mg/mL. Preliminary concentration optimisation assays were initially conducted at 0.25, 0.5, 1, and 2 mg/mL. Based on assay stability and inhibitory consistency, 0.25 mg/mL was selected for comparative analysis. The mixture was incubated for 25 min at a temperature of 37 °C, followed by adding 20 µL of the pNPB substrate solution. The final concentration of DMSO in the reaction mixture was maintained below 1% to minimise solvent interference with enzyme activity. The negative control was prepared by replacing the plant extract with 70% methanol using the same volume while blank prepared without plant extracts and enzymes solution. Orlistat (≥ 98% purity, Sigma-Aldrich, St. Louis, MO, USA) at a concentration of 20 µg/mL was used as a positive control. All assays were performed in triplicate, and absorbance values were expressed as mean ± standard deviation. The absorbance was read at 412 nm using a microplate spectrophotometer. Inhibition of pancreatic lipase was expressed as a percentage of inhibition and calculated using equation (Eq 1).

1 $$Pancreatic\:lipase\:inhibition\left(\%\right)=\left[\frac{Abscontrol-Abssample}{Abscontrol}\right]\times100$$

### Alpha-amylase inhibitory activity of plant extracts

The α-amylase (EC 3.2.1.1) inhibitory activity was determined using a modified method described by Buchholz et al. [24]. A 5% starch solution (w/v) was prepared by boiling potato starch in 20mM potassium phosphate buffer, pH 6.8. The α-amylase enzyme (1 U/mL) was also prepared by dissolving it in potassium phosphate buffer. Plant extracts were prepared at a concentration of 0.25 mg/mL using 70% methanol as the solvent. Initial concentration screening was performed at 0.25, 0.5, 1, and 2 mg/mL to evaluate inhibitory responses across different extract concentrations. Based on the stability and consistency of the inhibitory activity observed, 0.25 mg/mL was selected for subsequent comparative analyses. The reaction mixture was set up in a 96-well plate by adding 25 µL of α-amylase solution, 50 µL of the plant extract, and 25 µL of starch solution to start a reaction. The solution was incubated for 30 min at 37 °C. Thereafter, 50 µL of 96 mmol/L 3,5- dinitro salicylic acid solution was added and placed on a 100 °C water bath for 5 min. The control without plant extract was prepared, whereas 2 mM acarbose (≥ 98% purity, Sigma-Aldrich, St. Louis, MO, USA) was used as a positive control. The absorbance was read at 540 nm using a UV spectrophotometer. Inhibition of α-amylase was expressed as a percentage of inhibition and calculated by the following Eq. 2.

2 $$\alpha-amylase\:inhibition\left(\%\right)=\left[\frac{Abscontrol-Abssample}{Abscontrol}\right]\times100$$

### Alpha-glucosidase inhibitory activity of plant extracts

Alpha-glucosidase (EC 3.2.1.20) inhibition was determined using a modified method as described by Unuofin et al. [25]. The α-glucosidase enzyme from Saccharomyces cerevisiae (0.01 mg/mL) was prepared by dissolving in 20mM potassium phosphate buffer (pH 6.8). The 4 mM p-Nitrophenyl-α-D-glucopyranoside (pNPG) as a substrate was prepared in potassium phosphate buffer. Plant extracts were prepared by dissolving 0.25 mg/mL in 70% methanol. Preliminary concentration optimisation assays were initially conducted at 0.25, 0.5, 1, and 2 mg/mL. Based on assay stability and inhibitory consistency, 0.25 mg/mL was selected for comparative analysis. The reaction mixture was set up in a 96-well plate by adding 20 µL of α-glucosidase solution, followed by 20 µL of plant extract and 135 µL of potassium phosphate buffer. Thereafter, the mixture was incubated for 10 min at 37 °C, followed by the addition of 25 µL of pNPG. The mixture was allowed to stand for 20 min at a temperature of 37 °C. The control was prepared by adding 70% methanol instead of the plant extract, and 2 mM acarbose (≥ 98% purity, Sigma-Aldrich, St. Louis, MO, USA) was used as the positive control. The absorbance was recorded at 405 nm using a spectrophotometer. The inhibition percentage was calculated using Eq. 3.

3 $$\:\alpha\:-glucosidase\:inhibition\:\left(\%\right)=\left[\frac{Abscontrol-Abssample}{Abscontrol}\right]\times\:100$$

### Liquid chromatography-mass spectrometry (LCMS) based metabolite profiling

The metabolite profiling was performed using a high-resolution Waters Cyclic Quadrupole time-of-flight (qTOF) mass spectrometer (MS) coupled to a Waters Acquity ultra-performance liquid chromatograph (UPLC) systems (Waters, Milford, MA, USA). The qTOF platform was selected due to its high mass accuracy, sensitivity, and ability to simultaneously acquire precursor and fragmentation ion data, making it suitable for untargeted metabolomic profiling and relative comparison of metabolites across plant extracts [26]. An untargeted metabolomics approach was employed to facilitate broad-spectrum detection and comparative profiling of metabolites present in the extracts without prior selection of target compounds. The analytical workflow was executed under automated and independently controlled acquisition conditions, enabling simultaneous collection of spectral data. The column effluent was directed sequentially through a photodiode array (PDA) detector before MS detection, enabling concurrent acquisition of UV–visible and mass spectrometric data. The electrospray ionisation was performed in a positive ion mode of electrospray utilized a cone voltage of 15 V, a desolvation temperature of 275 °C, and a desolvation gas flow rate of 650 L/h. The remaining MS parameters were autonomously optimised by the instrument control software to ensure high mass accuracy, resolution, and sensitivity. Data were acquired over an m/z range of 100 to 1500 in a resolution mode and mass spectrometry elevated energy (MSE) mode, facilitating comprehensive metabolite detection. The MSE acquisition mode enabled simultaneous acquisition of precursor and fragment ion information in a single analytical run, thereby facilitating structural characterisation of detected metabolites. In MSE mode, data were collected simultaneously across two energy channels: a low-energy function at 4 V and a high-energy function ramped from 40 to 100 V, enabling automated acquisition of precursor and fragment ion information in a single analytical run. Accurate mass correction was achieved through continuous lock-mass calibration using leucine enkephalin, while external calibration was performed with sodium formate.

Chromatographic separation was obtained using a Waters HSS T3 column (2.1 × 150 mm, 1.7 μm). The 0.5 µL sample injection was performed using 0.1% formic acid in water as solvent A and followed by 0.5 µL acetonitrile containing 0.1% formic acid as solvent B. The gradient was programmed and initiated at 100% solvent A for 1 min, followed by a linear decrease to 28% solvent B over 11 min. The composition was then increased to 40% solvent B over 50 s, followed by a wash step at 100% solvent B for 1.5 min, and finally re-equilibrated to the initial conditions for 2 min. The flow rate was maintained at 0.3 mL/min, and the column temperature was kept constant at 60 °C. Metabolite annotation was achieved by comparing accurate mass measurements, fragmentation patterns, and retention time information with data available in online metabolite databases and previously published literature, including METLIN, MassBank, and PubChem databases [27]. Compound identification was therefore regarded as putative annotation based on Level 2 metabolite identification according to the Metabolomics Standards Initiative (MSI) guidelines. Chromatographic reproducibility and analytical stability were monitored throughout the analysis using routine instrument calibration and lock-mass correction procedures. Relative quantification of detected compounds was performed using an independently generated calibration curve constructed from rutin standards ranging from 0.2 to 5 mg/L, ensuring analytical consistency and reproducibility across runs. Rutin was used as a representative phenolic standard for relative quantification purposes and not for absolute quantification of structurally diverse metabolites. Therefore, metabolite concentrations reported in the present study should be interpreted as relative abundance estimates.

### Experimental design

The experiment was arranged in a factorial completely randomised design involving two plant species, namely *H. hemerocallidea* and *P. prunelloides.* Each plant species consisted of two treatments, including plant leaves and roots, with three independent replicates per treatment, resulting in a total of 12 experimental units. Furthermore, each replicate was measured three times to improve analytical accuracy.

### Statistical analysis

The statistical analyses were carried out using Statistical Computing Environment, R software, version 4.4.0 on the R Studio platform (Posit Software, PBC, 250 Northern Ave Suite 420 Boston, MA 02210, USA). The analysis of variance (ANOVA) was conducted to assess significant differences among sample means at a 5% significance level. Where significant differences were detected, Duncan’s Multiple Range Test (DMRT) was applied to separate group means. Multivariate analysis, including correlation and principal component analysis (PCA) were also performed in R Studio. The corrplot package was used to visualise correlation matrices, while FactoMineR (version 2.11) was used for PCA computation and interpretation. Correlation analyses were interpreted cautiously due to the exploratory nature of the study and the relatively limited sample size. Heat maps were initially constructed using MetaboAnalyst and subsequently modified and refined in R for improved visualization and presentation.

## Results

### Enzymatic inhibitory activity

The tested plant extracts from the roots and leaves of *H. hemerocallidea* and *P. prunelloides* demonstrated statistically significant inhibitory activity (*p* < 0.001) against digestive enzymes (Table 1). Comparative analysis between plant species revealed highly significant differences in pancreatic lipase inhibition (*p* < 0.001), whereas no significant differences were observed for α-glucosidase and α-amylase inhibition. Overall, *H. hemerocallidea* demonstrated higher pancreatic lipase inhibitory activity (82.51%) compared to *P. prunelloides* (38.94%).

**Table 1:** Inhibitory activity (%) of plant extracts from *H. hemerocallidea* and *P. prunelloides* on digestive enzymes at 0.25 mg/mL. Data expressed as mean ± standard deviation (*n* = 3)

Extract	α-Amylase inhibition (%)	α-Glucosidase inhibition (%)	Pancreatic lipase inhibition (%)
*P. prunelloides* roots	50.63^a^ ± 4.75	99.02^a^ ± 0.57	36.30^c^ ± 2.49
*P. prunelloides* leaves	35.56^b^ ±- 1.92	96.38^b^ ± 1.49	41.58^c^ ± 3.02
*H. hemerocallidea* roots	53.14^a^ ± 1.45	68.65^c^± 1.21	66.34^b^ ± 3.48
*H. hemerocallidea* leaves	11.71^c^ ± 3.63	98.10^ab^ ± 0.93	98.68^a^ ± 3.02
Positive control	55.65^a^ ± 1.45 (Acarbose)	99.94^a^ ± 0.11 (Acarbose)	96.37^a^ ± 4.54 (Orlistat)
LSD (PS*PP)	6.06	2.07	5.70
P. value (PS*PP)	<. 001	<. 001	<. 001

The values are presented as means (*n* = 3), the level of significant difference at *p* < 0.001 was observed on the leaves and roots of plant species and among enzymatic activity. Means sharing the same letter within the same column are not significantly different, whereas means with different letters differ significantly

*Abbreviations*: PS*PP Interaction between plant species and plant part, *LSD* Least significant difference

Among all extracts, *H. hemerocallidea* leaves extract exhibited the strongest pancreatic lipase inhibition (98.68%), exceeding the standard lipase inhibitor orlistat (96.38%). The same extract also showed strong α-glucosidase inhibition (98.10%), closely comparable to the positive control acarbose (99.94%), but demonstrated relatively weak α-amylase inhibition (11.71%).

The analysis of variance further revealed a highly significant interaction (*p* < 0.001) between plant species and plant part for enzyme inhibitory activity. Among all extracts, *H. hemerocallidea* leaves showed a strong inhibitory effect against pancreatic lipase (98.68%), surpassing the standard lipase inhibitor orlistat (96.38%). Additionally, the same extract exhibited the strong α-glucosidase inhibition (98.10%), closely approximating the positive control acarbose (99.94%). However, its inhibitory effect against α-amylase was considerably lower (11.71%). In contrast, *H. hemerocallidea* roots showed higher α-amylase inhibition (53.14%) among all tested plant extracts, which was slightly below the standard inhibitor acarbose (55.65%) and exhibited moderate inhibition of α-glucosidase (68.65%) and lipase (66.34%). *P. prunelloides* roots showed a strong α-glucosidase inhibition (99.02%), nearly equivalent to the standard inhibitor acarbose, moderate α-amylase inhibition (50.63%) and lower pancreatic lipase inhibition (36.30%). Notably, *P. prunelloides* leaves demonstrated a strong α-glucosidase inhibition (96.38%), modest pancreatic lipase inhibition (41.58%) and lower α-amylase inhibition (35.56).

### Liquid chromatography-mass spectrometry analysis identified different metabolite profiles

The untargeted LCMS analysis discovered a total number of 187 metabolites across the leaves and roots of *H. hemerocallidea* and *P. prunelloides*. Among these, 32 major compounds metabolites belonging to different phytochemical classes including flavonoids, phenolic acids, proanthocyanidins, saponins, terpenoids, phenylethanoid glycosides, and xanthones were putatively identified based on accurate mass measurements, retention time, database annotation, and MS/MS fragmentation patterns (Table 2). The selected metabolites were associated with previously reported inhibitory activities against digestive enzymes involved in lipid and carbohydrate metabolism.

**Table 2:** Putatively identified metabolites detected in *Hypoxis hemerocallidea* and *Pentanisia prunelloides* extracts using untargeted LC–MS/MS analysis

Putatively identified metabolite	Average Rt(min)	Average Mz	Formula	Total score	Databases	Major MS/MS fragments (m/z)
Flavonoids						
Kaempferol 3-(6-acetylgalactoside)	5,75	489,10	C23H22O12	6,15	HMDB, KNApSAcK	255.03, 284.03, 489.10
quercetin 3-O-alpha-(6’’’-caffeoylglucosyl-beta-1,2-rhamnoside)	5,214	771,18	C36H36O19	6,44	HMDB, ChEBI	271.02, 300.03, 771.18
Rutin	4,775	609,15	C27H30O16	7,95	HMDB, ChEBI	325.09, 577.13, 609.15
Vitexin 2’’-O-rhamnoside-4’’’-acetate	4,653	619,17	C29H32O15	6,01	KNApSAck, HMDB	281.08, 295.06, 619.17
Tricin 7-glucoside	4,341	491,12	C23H24O12	6,58	KNApSAck, HMDB	279.07, 297.08, 459.13
5,7-Dimethoxyflavone	4,661	281,08	C17H14O4	4,84	KNApSAck, HMDB	147.03, 228.05, 278.02
5-hydroxy-7-methoxy-2-(4-methoxyphenyl)-4 H-chromen-4-one	4,703	297,08	C17H14O5	5,37	KNApSAck, HMDB	147.03, 228.05, 295.06
Lonicerin; Luteolin 7-neohesperidoside	4,173	593,15	C27H30O15	7,73	KNapSAck, PubChem	175.06, 379.16, 593.15
Pectolinarigenin 7-O-rutinoside	4,703	621,18	C29H34O15	5,93	KNApSAcK, ChEBI	147.03, 619.17, 621.18
6’’-O-p-Coumaroyltrifolin	3,423	593,13	C30H26O13	6,86	HMDB, PubChem	177.02, 305.07, 593.13
Embigenin 2’’-(2’’’-acetylrhamnoside)	5,762	647,19	C31H36O15	5,47	HMDB	281.08, 485.14, 647.20
6’’-O-Acetyldaidzin	5,347	457,11	C23H22O10	6,68	KNApSAck, HMDB,	151.04, 299.09, 457.11
Genistein	4,226	269,05	C15H10O5	5,53	HMDB, KNApSAcK	239.06, 240.06
Phenolic Acids						
Protocatechuic acid	3,149	153,02	C7H6O4	7,34	HMDB, KNApSAck	109.03, 153.02
Gentesic acid 5-O-glucoside	3,152	315,07	C13H16O9	6,49	PubChem	109.03, 153.02, 315.07
Salicylic acid	3,564	137,02	C7H6O3	6,99	KNApSAcK, HMDB	136.02, 137.02
Chlorogenic acid	4,033	353,09	C16H18O9	6,77	KNApSAcK, HMDB	191.06, 289.07, 353.09
Caffeic acid 4-O-glucuronide	3,695	355,07	C15H16O10	5,61	HMDB, FooDB	123.05, 255.05, 355.07
Proanthocyanidins / Flavan-3-ols						
(-)-Epigallocatechin	3,43	305,07	C15H14O7	7,19	KNApSAcK, HMDB	177.02, 303.05, 305.07
Procyanidin B5	3,735	577,14	C30H26O12	7,15	HMDB, ChEBI	177.02, 289.07, 577.14
Epicatechin	4,017	289,07	C15H14O6	6,69	KNApSAcK, HMDB	191.06, 287.06, 289.07
Saponins / Triterpenoids						
Atractyloside A 14-O-beta-D-fructofuranoside	5,994	655,28	C27H46O15	5,28	KNApSAcK, COCONUT	281.08, 609.28, 655.28
Vittarin D	4,054	479,16	C23H28O11	6,53	KNApSAcK, COCONUT	281.08, 315.09, 333.10
Terpenoids / Aromatic esters						
Centapicrin	4,801	519,15	C25H28O12	6,68	KNApSAcK, COCONUT	191.07, 477.20, 519.15
Linalool 3,7-oxide beta-primeveroside	4,855	463,22	C21H36O11	4,89	KNApSAcK, FooDB	283.10, 445.15, 463.22
Phenolic Glycosides						
Minecoside	4,407	537,16	C25H30O13	6,75	KNApSAcK, ChEBI	391.11, 479.15, 537.16
Acteoside	4,747	623,19	C29H36O15	6,98	HMDB, ChEBI	295.06, 603.17, 623.20
Plantamajoside	5,239	639,19	C29H36O16	5,89	KNApSacK, ChEBI	146.97, 639.19
Xanthones						
Polygalaxanthone X	3,613	639,19	C29H36O16	5,77	KNApSAcK, COCONUT	315.09, 477.14, 639.19
Mycophenolic acid O-acyl-glucuronide	3,869	495,15	C23H28O12	6,48	HMDB, ChEBI	161.06, 315.09, 495.15
1,7-Dihydroxy-2,3-methylenedioxyxanthone	4,777	271,03	C14H8O6	4,48	KNApSAcK, COCONUT	174.96
Julichrome Q6 glucuronide	4,702	519,15	C25H28O12	6,16	KNApSAcK, COCONUT	297.08, 459.13, 519.15

**Fig. 1:**
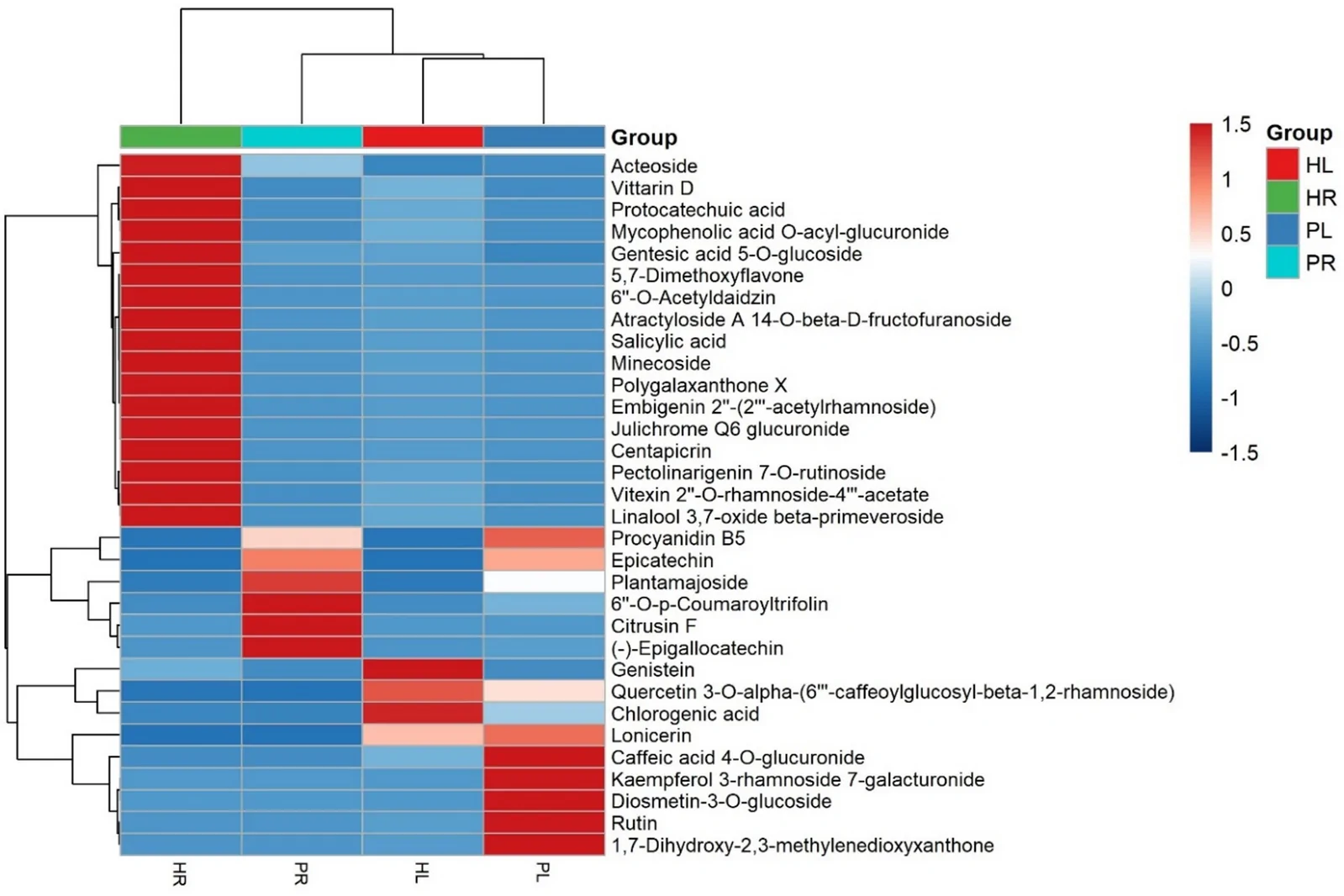
Heat maps illustrate the relative abundance of secondary metabolites detected in the methanolic extracts of *H. hemerocallidea* and *P. prunelloides* leaves and roots using LCMS. Metabolite clustering (rows) and sample grouping (columns) reveal distinct tissue and species-specific metabolite accumulation patterns. The red boxes indicate higher concentration, while the blue boxes indicate lower or zero concentration

The heatmap analysis revealed distinct clustering patterns among the different plant parts and species, indicating substantial metabolic variation between the extracts (Fig. 1). Flavonoids, including kaempferol 3-(6-acetylgalactoside), rutin, lonicerin, quercetin 3-O-(6’’’-caffeoylglucosyl-β-1,2-rhamnoside) were generally more abundant in the *P. prunelloides* leaves. The 5,7-dimethoxyflavone, Embigenin 2’’-(2’’’-acetylrhamnoside), vitexin 2’’-O-rhamnoside, and pectolinarigenin 7-rutinoside were predominantly detected in *H. hemerocallidea* roots. In contrast, phenolic acids, including chlorogenic acid accumulated in the leaf extract of *H. hemerocallidea* while caffeic acid 4-O-glucuronide, accumulated mainly in the leaf extracts of *P. prunelloides* leaves. The salicylic acid, protocatechuic acid, and gentisic acid 5-O-glucoside dominated in *H. hemerocallidea* roots.

Furthermore, phenolic glycosides, including acteoside, and minecoside, were highly concentrated in the *H. hemerocallidea* roots, while plantamajoside was higher in *P. prunelloides* roots. Mycophenolic acid O-acyl-glucuronide and centapicrin were also dominant in *H. hemerocallidea* roots. The proanthocyanidins such as (-) epigallocatechin, epicatechin and procyanidin B5 were predominated in roots of *P. prunelloides*. The saponins atractyloside A 14-O-β-D-fructofuranoside and vittarin D, and terpenoids such as linalool 3,7-oxide β-primeveroside and centapicrin were accumulated in roots of *H. hemerocallidea* while citrusin F dominated in *P. prunelloides* roots. The xanthone derivatives 1,7-Dihydroxy-2,3-methylenedioxyxanthone were higher in *P. prunelloides* leaves, while polygalaxanthone X abundance in *H. hemerocallidea* roots. The glucuronide conjugates include julichrome Q6 glucuronide and mycophenolic acid O-acyl-glucuronide accumulated in *H. hemerocallidea* roots.

### Correlation analysis between enzyme inhibitory activities and bioactive compounds

The correlation analysis was executed to explore the potential relationship between the inhibitory activities of key digestive enzymes and the relative abundance of individual phytochemical compounds. The analysis demonstrated strong negative correlation, particularly with α-glucosidase inhibitory activity, suggesting that increased abundance of certain metabolites may be associated with enhanced enzyme inhibition (Table 3). However, these associations should be interpreted cautiously due to the exploratory nature of the study and the limited sample size.

**Table 3:** Representative correlations between enzyme inhibitory activities and putatively identified bioactive metabolites detected in the root and leaves of *H*. *hemerocallidea* and *P. prunelloides*

Compound	Enzyme	Correlation (*r*)	*p*-value	Interpretation
Pectolinarigenin-7-O-rutinoside	α-Glucosidase	−0.994	0.006	*
Vitexin-O-rhamnoside acetate	α-Glucosidase	−0.990	0.010	*
5,7-Dimethoxyflavone	α-Glucosidase	−0.997	0.003	*
Embigenin acetylrhamnoside	α-Glucosidase	−0.996	0.004	*
X6-O-acetyldaidzin	α-Glucosidase	−0.996	0.004	*
Protocatechuic acid	α-Glucosidase	−0.986	0.014	*
Gentisic acid 5-O-glucoside	α-Glucosidase	−0.981	0.019	*
Salicylic acid	α-Glucosidase	−0.996	0.004	*
Acteoside	α-Glucosidase	−0.956	0.044	*
Centapicrin	α-Glucosidase	−0.996	0.004	*
Vittarin D	α-Glucosidase	−0.982	0.018	*
Atractyloside A	α-Glucosidase	−0.996	0.004	*
Linalool oxide	α-Glucosidase	−0.991	0.009	*
Polygalaxanthone X	α-Glucosidase	−0.996	0.004	*
Quercetin caffeoyl rhamnoside	α-Amylase	−0.968	0.032	*
Chlorogenic acid	α-Amylase	−0.989	0.011	*
Genistein	Pancreatic lipase	0.947	0.053	Strong trend
Epicatechin	Pancreatic lipase	−0.886	0.114	Strong trend
Procyanidin B5	Pancreatic lipase	−0.837	0.163	Strong trend
Plantamajoside	Pancreatic lipase	−0.843	0.157	Strong trend

Pearson correlation coefficients (r) and p-values showing the relationships between selected metabolites and digestive enzyme inhibitory activities. Significant negative correlations (*p* < 0.05) indicate a strong association between compound abundance and enzyme inhibitory activity. Compounds marked as “strong trend” suggest potential bioactivity, though not statistically significant

_*_, indicate statistically significant difference

Several metabolites showed strong and significant negative correlation with α-glucosidase inhibitory activity (*r* = − 0.956 to − 0.997; *p* < 0.05). These included flavonoid glycosides and aglycones such as pectolinarigenin-7-O-rutinoside, vitexin-O-rhamnoside acetate, 5,7-dimethoxyflavone, and embigenin acetylrhamnoside. Additionally, strong negative correlation were also observed for phenolic and glycosylated derivatives including X6-O-acetyldaidzin, protocatechuic acid, gentisic acid 5-O-glucoside, salicylic acid, acteoside, and centapicrin. Further, vittarin D, atractyloside A, linalool oxide, and polygalaxanthone X, exhibited similarly strong negative correlations with α-glucosidase inhibitory activity (*r* = − 0.98 to 0.99; *p* < 0.05).

The α-amylase inhibitory activity demonstrated strong and significant negative correlations with quercetin caffeoyl rhamnoside and chlorogenic acid (*r* = − 0.968 and − 0.989; *p* < 0.05) respectively. In contrast, pancreatic lipase inhibitory activity showed no statistically significant correlation (*p* > 0.05) although several metabolites displayed a notable correlation trend. Genistein exhibited a strong positive correlation with lipase inhibitory activity (*r* = 0.947), while epicatechin, procyanidin B5, and plantamajoside exhibited strong negative trends (*r* = − 0.837 to − 0.886). These observations suggest potential associations between metabolite abundance and enzyme inhibitory activity and provide a preliminary foundation for future targeted validation studies.

### Principal component analysis of digestive enzymes, plant parts and metabolites

**Fig. 2:**
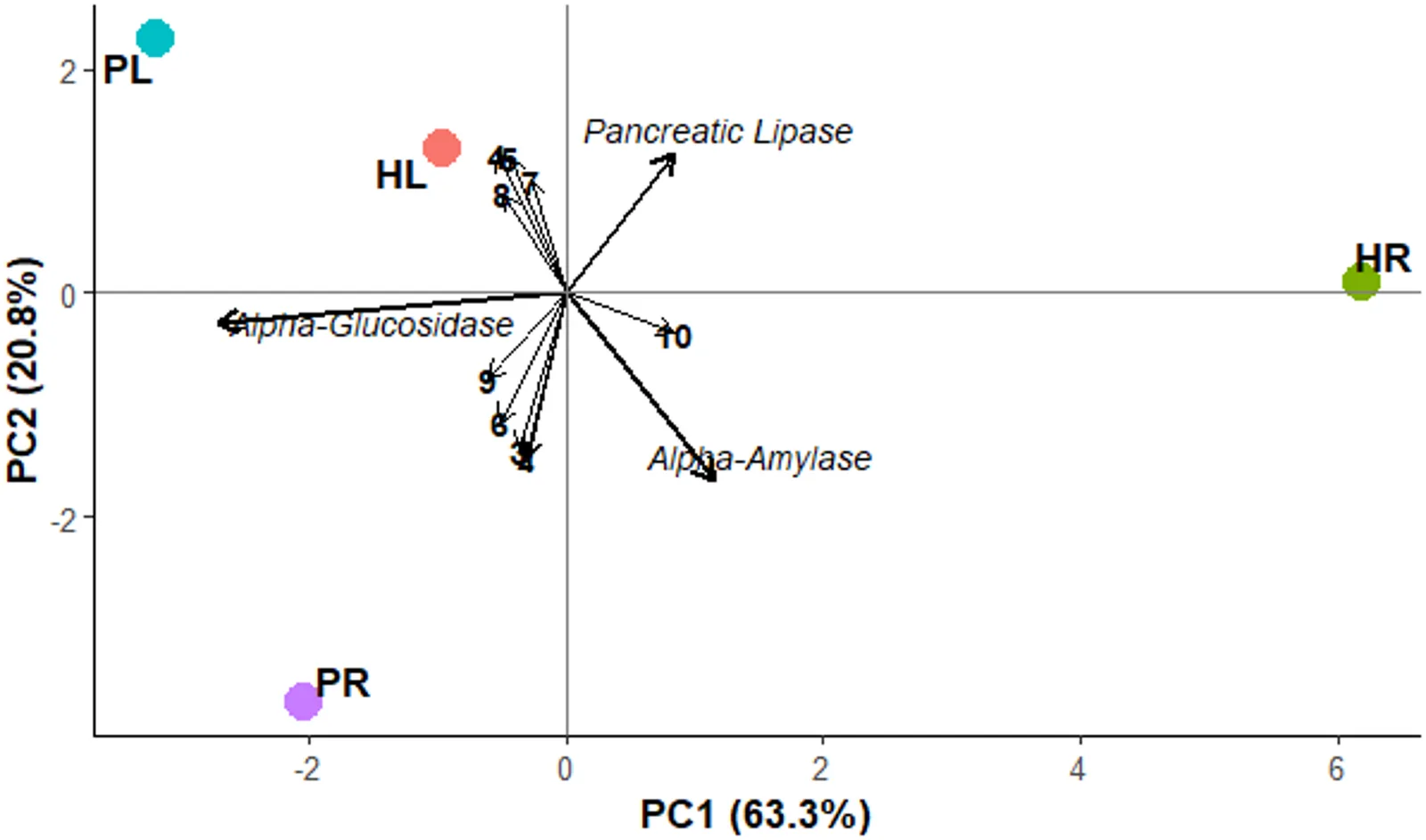
PCA biplot showing the relationship between digestive enzyme inhibitory activity and major metabolites in *Hypoxis hemerocallidea* and *Pentanisia prunelloides* extracts. The biplot represents the sample score (extracts), enzyme activity vectors, and the top ten phytochemical compounds based on their loading magnitudes. The values are presented as means (*n* = 3) and LSD (*n* = 12). Abbreviations: PL, *Pentanisia prunelloides* leaves; PR, *Pentanisia prunelloides* roots; HL, *Hypoxis hemerocallidea* leaves; HR, *Hypoxis hemerocallidea* roots; 1, Citrusin F; 2, Epigallocatechin; 3, 6-O-p-coumaroyltrifolin; 4, Lonicerin; 5, Quercetin-caffeoyl-rhamnoside; 6, Plantamajoside; 7, Chlorogenic acid; 8, Caffeic acid-4-O-glucoside; 9, Epicatechin; 10, Acteoside

PCA illustrates the multivariate distribution of enzymatic activity in relation to the ten metabolites contributing most to the separation of roots and leaves extract of *H. hemerocallidea* and *P. prunelloides* (Fig. 2). Principal components and corresponding correlation coefficient (eigenvectors) for the plant parts and metabolites parameters were grouped in Table 3. The first two principal components explained 84.1% of the total variance, indicating that the biplot adequately represents the underlying data structure, PC1 accounting for 63.3% and PC2 accounting for 20.8%.

The *H. hemerocallidea* roots clustered on the positive axis of PC1, reflecting a distinct metabolites profile compared to the other extracts. In contrast, the leaves and roots of *P. prunelloides* and *H. hemerocallidea* leaves were clustered on the negative side of PC1, indicating the phytochemical similarity among these samples. Additional variation was observed along PC2, where the leaves of *P. prunelloides* and *H. hemerocallidea* were positioned in the positive region, while *P. prunelloides* roots positioned in the negative quadrant, suggesting variation in metabolite abundance patterns among the extracts.

The direction and magnitude of the enzyme vectors indicated the relationships among the measured variables. Pancreatic lipase inhibitory activity demonstrated a strong positive loading on PC2 and a moderate relationship with PC1, suggesting that metabolites clustered along this axis may be associated with lipase inhibitory activity. The observation showed that α-amylase and α-glucosidase vectors were oriented more closely along PC1, reflecting that variation along this component was more strongly associated with carbohydrate-digesting enzyme inhibitory activity.

Several metabolites were positioned more closely to the enzyme vectors, suggesting potential associations with inhibitory activity. Chlorogenic acid (7) and caffeic acid-4-O-glucoside (8**)** were clustered near the pancreatic lipase vector, indicating a stronger correlation with lipase inhibitory activity and highlighting the possible involvement of phenolic acids in lipid digestion regulation. Acteoside (10) and epicatechin (9) were clustered in proximity with the α-amylase and α-glucosidase vectors, indicating a strong association with carbohydrate-hydrolysing enzyme inhibitory activity. Lonicerin (4) and quercetin-caffeoyl-rhamnoside (5) were located in the upper region of PC2, indicating possible associations with pancreatic lipase inhibitory activity and weaker associations with α-glucosidase inhibition. In contrast, epigallocatechin (2) and 6-O-p-coumaroyltrifolin (3) were oriented in the lower PC2 region, suggesting a weaker association with pancreatic lipase inhibitory activity. Furthermore, citrusin F (1) and plantamajoside (6) demonstrated limited proximity to enzyme vectors, indicating minor or indirect associations with enzyme inhibitory activity Table 4.

**Table 4:** PCA factor scores (plant parts means) and compound loadings (eigenvectors) for the first two principal components (PC1 and PC2). PC1 and PC2 explain 63.3% and 20.8% of the total variance, respectively, with a cumulative variance of 84.1%

Factor (Factor score)	PC1	PC2
HL	−0.95	1.28
HR	6.19	0.09
PR	−2.04	−3.65
PL	−3.20	2.27

Loadings (Eigenvector)	PC1	PC2
Citrusin F	−0.08	−0.38
Epigallocatechin	−0.09	−0.38
6-O-p-coumaroyltrifolin	−0.10	−0.36
Lonicerin	−0.14	0.33
Quercetin-caffeoyl-rhamnoside	−0.11	0.29
Plantamajoside	−0.14	−0.26
Chlorogenic acid	−0.05	0.16
Caffeic acid-4-O-glucoside	−0.13	0.28
Epicatechin	−0.15	−0.13
Acteoside	0.20	−0.04
Proportion of total Variance (%)	63.3%	20.8%
Cumulative (%)	63.3%	84.1%

*Abbreviations*: *PC* Principal component, *HL* Hypoxis hemerocallidea leaves, *HR* Hypoxis hemerocallidea roots, *PR* Pentanisia prunelloides roots, *PL* Pentanisia prunelloides leaves

## Discussion

The observed results demonstrated that *H. hemerocallidea*, and *P. prunelloides* extracts possess a significant inhibitory activity against key digestive enzymes involved in carbohydrate and lipid digestion, suggesting their potential relevance in the management of obesity-related metabolic disorders. Additionally, the enzyme inhibitory activity varied between plant species and plant parts, indicating that enzyme-specific and tissue-specific factors influence bioactivity. These inhibitory activities were closely associated with the phytochemical composition and relative abundance of metabolites, as evidenced by metabolomic profiling, which identified various compound classes including flavonoids, phenolic acids, phenolic glycosides, proanthocyanidins, saponins, terpenoids, and xanthones. These findings are consistent with those reported by Mngoma et al. [18], who demonstrated that extracts from *H. hemerocallidea* and *P. prunelloides* are rich in phenolic compounds and exhibit strong antioxidant activity. The majority of these compounds have previously been reported to inhibit lipid and carbohydrate digestion and absorption by reducing enzymatic activity in a concentration-dependent manner through their structural features such as glycosylation, hydroxylation, and conjugation [28].

*H. hemerocallidea* leaves demonstrated strong pancreatic lipase (98.68%) and α-glucosidase (98.10%) inhibitory activities and were particularly rich in chlorogenic acid and caffeic acid-4-O-glucuronide. These compounds have previously been reported as lipase inhibitors through interactions with catalytic residues involved in lipid digestion [29]. Similarly, Raghavendra et al. [30] reported that chlorogenic acid and caffeic acid inhibited rice bran lipase, through engaging in hydrogen bonding and hydrophobic interactions that interfere with enzymatic activity. The PCA biplot analysis further demonstrated that these metabolites clustered near the pancreatic lipase inhibitory activity vector, suggesting a potential association with lipase inhibitory activity. The same extract demonstrated weak α-amylase inhibition (11.71%). This selectivity may be therapeutically beneficial by targeting postprandial hyperlipidemia and hyperglycemia while potentially minimising gastrointestinal adverse effects associated with excessive amylase inhibition. However, the physiological relevance of these in vitro observations requires further validation using in vivo and mechanistic studies. In contrast, *H. hemerocallidea* roots exhibited higher α-amylase inhibition (53.14%), were enriched with various metabolites including xanthones (polygalaxanthone X), acetylated flavonoids (5,7-dimethoxyflavone), and phenolic glycosides such as acteoside. These metabolites have previously been associated with inhibitory effects against starch-degrading enzymes through competitive binding or structural disruption of active sites [28]. Similarly, Mahlo et al. [31] reported inhibitory activities against α-amylase and α-glucosidase in different solvent fractions of *H. hemerocallidea*, supporting the enzyme inhibitory potential of this species. However, variations in inhibitory activity between studies may be attributed to differences in extraction methods, phytochemical composition, plant parts, and experimental conditions.

The *P. prunelloides* leaves and roots, exhibited strong α-glucosidase inhibitory activity (96.38%; 99.02% respectively, ) and were characterised by higher relative abundance of flavonoids, including kaempferol 3-(6-acetylgalactoside), rutin, lonicerin, and quercetin 3-O-(6’’’-caffeoylglucosyl-β-1,2-rhamnoside), particularly in the leaves. These flavonoids have been reported to inhibit α-glucosidase through strong binding affinity within the catalytic pocket, particularly via hydroxyl substitutions at the C3 and C5 positions and sugar moieties that improve solubility and enzyme-binding affinity [32]. For example, quercetin derivatives have been reported to interact with key residues in the active site of α-glucosidase, interfering with the hydrolysis of disaccharides into absorbable glucose [33]. The PCA analyses further revealed that several of these flavonoids clustered near the α-glucosidase inhibitory activity vector, suggesting potential associations between their abundance and enzyme inhibitory activity. Similarly, Makhubu et al. [34] reported strong α-glucosidase inhibitory activities in *P. prunelloides* root extracts, accompanied by relatively high total flavonoid content, further supporting the possible contribution of flavonoids to carbohydrate-digesting enzyme inhibition.

The *P. prunelloides* roots and leaves exhibited relative higher abundance of proanthocyanidins, particularly epicatechin and procyanidin B5, together with inhibitory activity against both α-glucosidase and pancreatic lipase. Similarly, You et al. [35] reported that cyanidin extracted from grapes inhibited both α-glucosidase and pancreatic lipase. The inhibitory activities may be attributed to their polymeric structure and strong antioxidant properties of proanthocyanidins, which have been reported to form non-covalent interactions with digestive enzymes and alter their tertiary structure, thereby affecting catalytic activity [36]. Although correlations with pancreatic lipase inhibitory activity were not statistically significant, the observed trends may indicate potential combined contributions of multiple phenolic compounds within the crude extracts.

Several glycosylated phenolic acids, including protocatechuic acid, gentisic acid 5-O-glucoside, and salicylic acid, were dominant in the roots of *H. hemerocallidea* and also demonstrated a strong negative correlation with α-glucosidase inhibitory activity (*p* < 0.05). These observations suggest potential associations between metabolite abundance and enzyme inhibitory activity and may provide a basis for future targeted validation studies.

Overall, the observed enzyme inhibitory activities may not necessarily be attributed to a single phytochemical compound, but rather to the combined effects of multiple metabolites present in the crude extracts. The qTOF-MS profiling revealed the presence of several compounds previously associated with antioxidant and enzyme inhibitory properties. Therefore, possible synergistic or additive interactions among these metabolites may have contributed to the overall bioactivity observed. However, further studies involving compound isolation and individual activity validation are required to confirm these interactions and identify the major bioactive constituents responsible for the observed effects.

## Conclusion

The study revealed that *H. hemerocallidea* and *P. prunelloides* are rich sources of secondary metabolites associated with inhibitory activities against key digestive enzymes involved carbohydrate and lipid metabolism. The strong inhibitory activities of the enzyme were observed, particularly from *H. hemerocallidea*, suggesting its relevance in the modulation of lipid and carbohydrate digestion. Metabolomic profiling revealed the presence of several phytochemical classes, including flavonoids, phenolic acids, phenolic glycosides, proanthocyanidins, saponins, terpenoids, and xanthones which have previously been associated with digestive enzyme inhibitory activity. The integration of untargeted metabolomic profiling with functional enzyme assays provided preliminary insights into metabolite and enzyme associations in these medicinal plants. Despite, several metabolites demonstrated strong associations with enzyme inhibitory activities, no compound isolation or activity-guided validation was performed in the present study. Furthermore, no cytotoxicity, safety, and in vivo evaluations were conducted. Therefore, the findings should be considered preliminary and require further pharmacological, toxicological, and mechanistic validation to confirm the therapeutic relevance of these extracts in obesity-associated metabolic disorders.

## Supplementary Information

Supplementary Material 1.

## Data Availability

The datasets used and analysed during the current study are available from the corresponding author on reasonable request.
