# Supplementary material for: In vitro inhibitory effects of Hypoxis hemerocallidea and Pentanisia prunelloides on α-amylase, α-glucosidase and pancreatic lipase activities associated with obesity-related metabolic disorders
Source: BMC Complement Med Ther. 2026 Jul 18;26:297. doi: 10.1186/s12906-026-05458-4 (PMC13663055; doi:10.1186/s12906-026-05458-4)
Supplement: Supplementary file 1 [file 12906_2026_5458_MOESM1_ESM.docx]

Table S1. Effect of plant extract and concentration on α-amylase, α-glucosidase, and pancreatic lipase inhibitory activities of Hypoxis hemerocallidea and Pentanisia prunelloides extracts.

| **Extract** | **Concentration (mg/mL)** | **α-Amylase inhibition (%)** | **α-Glucosidase inhibition (%)** | **Pancreatic lipase inhibition (%)** |
| --- | --- | --- | --- | --- |
| *P. prunelloides* roots | 0.25 | 50.63ab ± 4.75 | 99.02a ± 0.57 | 36.30cd ± 2.49 |
|  | 0.5 | 47.28abc ± 1.26 | 98.28ab ± 0.28 | 16.83de ± 7.53 |
|  | 1 | 43.10bc ± 3.14 | 96.14abc ± 2.20 | −26.31fg ± 11.50 |
|  | 2 | 25.10de ± 8.92 | 95.83abc ± 0.37 | −119.14i ± 27.67 |
|  |  |  |  |  |
| *P. prunelloides* leaves | 0.25 | 35.56cd ± 1.92 | 96.38abc ± 1.49 | 41.58c ± 3.02 |
|  | 0.5 | 12.97ef ± 1.90 | 95.34abc ± 0.53 | 49.84bc ± 12.00 |
|  | 1 | −2.37gh ± 1.14 | 76.38e ± 13.11 | 0.34ef ± 49.54 |
|  | 2 | −68.84j ± 2.17 | 83.62d ± 3.06 | −68.72h ± 28.75 |
|  |  |  |  |  |
| *H. hemerocallidea* roots | 0.25 | 53.14a ± 1.45 | 68.65f ± 1.21 | 66.34b ± 3.48 |
|  | 0.5 | 41.00bc ± 0.00 | 56.63g ± 1.10 | 57.10bc ± 2.63 |
|  | 1 | 40.17bc ± 4.40 | 52.02h ± 0.49 | 38.94cd ± 3.03 |
|  | 2 | 31.38d ± 7.15 | 38.16i ± 1.01 | −108.25i ± 66.65 |
|  |  |  |  |  |
| *H. hemerocallidea* leaves | 0.25 | 11.71f ± 3.63 | 98.10ab ± 0.93 | 98.68a ± 3.02 |
|  | 0.5 | 4.32fg ± 4.18 | 96.66abc ± 0.28 | 95.38a ± 12.92 |
|  | 1 | −46.91i ± 10.22 | 93.07c ± 0.00 | 86.80a ± 18.48 |
|  | 2 | −87.79k ± 58.26 | 89.14cd ± 0.70 | 35.30cd ± 45.23 |
|  |  |  |  |  |
| Positive control |  | 55.65a ± 1.45 (Acarbose) | 99.94a ± 0.11 (Acarbose) | 96.37a ± 4.54 (Orlistat) |
| LSD (E * C) |  | 12.54 | 4.37 | 31.82 |
| P-value (E * C) |  | < 0.001 | < 0.001 | < 0.001 |

Abbreviations: E*C, interaction between Extract and concentration; LSD, least significant difference. Values are presented as mean ± standard deviation (n = 3), the level of significant difference at *p* < 0.001 was observed on the leaves and roots of plant species and among enzymatic activity. Means of treatments sharing the same letter (s) within each enzymatic inhibitory activity were not significantly different at 5% level of significance.
